# In a Model of Batten Disease, Palmitoyl Protein Thioesterase-1 Deficiency Is Associated with Brown Adipose Tissue and Thermoregulation Abnormalities

**DOI:** 10.1371/journal.pone.0048733

**Published:** 2012-11-06

**Authors:** Alfia Khaibullina, Nicholas Kenyon, Virginia Guptill, Martha M. Quezado, Li Wang, Deloris Koziol, Robert Wesley, Pablo R. Moya, Zhongjian Zhang, Arjun Saha, Anil B. Mukherjee, Zenaide M.N. Quezado

**Affiliations:** 1 The Sheikh Zayed Institute for Pediatric Surgical Innovation, Children’s National Medical Center, Washington District of Colmbia, United States of America; 2 Department of Perioperative Medicine, NIH Clinical Center, National Institutes of Health, Bethesda, Maryland, United States of America; 3 Laboratory of Pathology, National Cancer Institute, National Institutes of Health, Bethesda, Maryland, United States of America; 4 Biostatistics and Clinical Epidemiology Service, Office of the Director, NIH Clinical Center, National Institutes of Health, Bethesda, Maryland, United States of America; 5 Laboratory of Clinical Science, National Institute of Mental Health, National Institutes of Health, Bethesda, Maryland, United States of America; 6 Section on Developmental Genetics, Program on Developmental Endocrinology and Genetics, Eunice Kennedy Shriver, National Institute of Child Health and Human Development, National Institutes of Health, Bethesda, Maryland, United States of America; Lousiana State University Health Sciences Center, United States of America

## Abstract

Infantile neuronal ceroid lipofuscinosis (INCL) is a fatal neurodegenerative disorder caused by a deficiency of palmitoyl-protein thioesterase-1 (PPT1). We have previously shown that children with INCL have increased risk of hypothermia during anesthesia and that PPT1-deficiency in mice is associated with disruption of adaptive energy metabolism, downregulation of peroxisome proliferator-activated receptor γ coactivator 1α (PGC-1α), and mitochondrial dysfunction. Here we hypothesized that *Ppt1*-knockout mice, a well-studied model of INCL that shows many of the neurologic manifestations of the disease, would recapitulate the thermoregulation impairment observed in children with INCL. We also hypothesized that when exposed to cold, *Ppt1*-knockout mice would be unable to maintain body temperature as in mice thermogenesis requires upregulation of *Pgc-1α* and uncoupling protein 1 (*Ucp-1*) in brown adipose tissue. We found that the *Ppt1*-KO mice had lower basal body temperature as they aged and developed hypothermia during cold exposure. Surprisingly, this inability to maintain body temperature during cold exposure in *Ppt1*-KO mice was associated with an adequate upregulation of *Pgc-1α* and *Ucp-1* but with lower levels of sympathetic neurotransmitters in brown adipose tissue. In addition, during baseline conditions, brown adipose tissue of *Ppt1*-KO mice had less vacuolization (lipid droplets) compared to wild-type animals. After cold stress, wild-type animals had significant decreases whereas *Ppt1*-KO had insignificant changes in lipid droplets compared with baseline measurements, thus suggesting that *Ppt1*-KO had less lipolysis in response to cold stress. These results uncover a previously unknown phenotype associated with PPT1 deficiency, that of altered thermoregulation, which is associated with impaired lipolysis and neurotransmitter release to brown adipose tissue during cold exposure. These findings suggest that INCL should be added to the list of neurodegenerative diseases that are linked to alterations in peripheral metabolic processes. In addition, extrapolating these findings clinically, impaired thermoregulation and hypothermia are potential risks in patients with INCL.

## Introduction

Neuronal ceroid lipofuscinoses (NCLs), also known as Batten disease [1], [2], [3], [4], represent a group of the most common hereditary neurodegenerative diseases in children. Among NCLs, the infantile subtype (INCL) has one of the earliest ages of onset, is relentlessly progressive, and is the most lethal neurodegenerative storage disorder in children. INCL has an incidence of 1 in >100,000 births and an autosomal recessive pattern of transmission. Children with INCL appear normal at birth, begin to develop psychomotor retardation, myoclonus, and seizures by 11–18 months, most develop blindness by 24 months, and most have complete loss of cortical cerebral function by 4 years of age. These children then live in a vegetative state for a few more years and succumb to the disease by late childhood [1], [2], [3], [4]. Given this presentation and lack of disease-altering therapy, there is a need for further understanding the pathogenesis of INCL.

**Figure 1:**
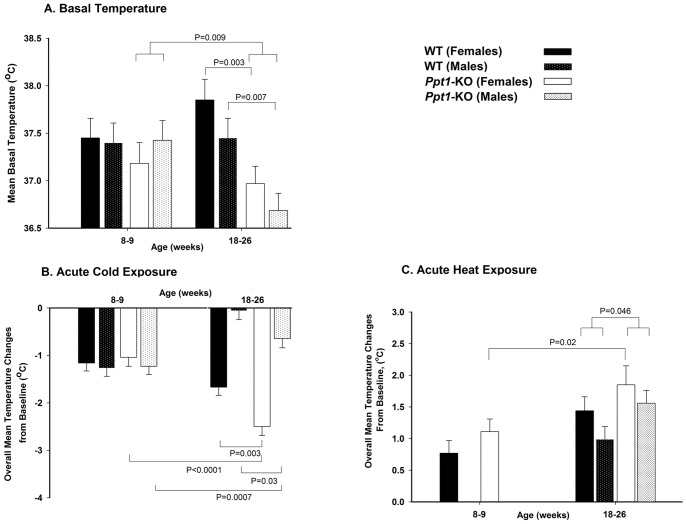
Body temperature during basal conditions and cold and heat exposures in *Ppt1*-KO and wild-type mice. In all panels, horizontal bars and respective p values show comparisons among groups indicated. A. Longitudinal measurements (least square mean ±SE) of basal temperature of male and female *Ppt1*-KO and wild-type mice at 8–9 and 18–26 weeks (N = 10 in each group). B. Temperature changes from baseline measured every 30 min during a 6-h (all time points combined) acute cold exposure at young (8–9 weeks) and old (18–26 weeks) ages. Bars represent least square means ±SE. C. Temperature changes (least square means ±SE) from baseline measured (every 30 min) during a 6-hour (all time points combined) acute heat exposure at young (8–9 weeks) and old (18–26 weeks) ages. In acute cold and heat exposure experiments, N = 5 in each group.

The genetic bases of INCL are inactivating mutations in the gene that encodes for palmitoyl-protein thioesterase 1 (PPT1). PPT1 is a lysosomal enzyme that cleaves thioester linkages in S-acylated (palmitoylated) proteins and facilitates the degradation of fatty-acylated proteins by lysosomal hydrolases [5], [6]. A deficiency of PPT1, in turn, leads to abnormal lysosomal accumulation of palmitoylated proteins in several cell types [7], excessive endoplasmic reticulum- and oxidative stresses causing apoptosis [8], [9], [10], neuroinflammation [11] and impairment of synaptic vesicle recycling [12]. All these effects of PPT1 deficiency appear to play a definite role in the cellular and molecular mechanisms underlying the progressive neurodegenerative process in INCL. Another contributing factor for neurodegeneration in INCL is the disruption of adaptive energy metabolism. This notion is supported by findings that fibroblasts from INCL patients and brain tissue from mice lacking PPT1 have significantly decreased levels of the peroxisome proliferator-activated receptor-γ coactivator-1α (PGC-1α) and of NAD-dependent deacetylase sirtuin 1 [13]. Therefore, similar to what has been shown in other neurologic disorders such as Huntington [14], Parkinson [15], and Alzheimer [16] diseases, adaptive energy metabolism and mitochondrial dysfunction appear to play a role in neurodegeneration in INCL.

Children with INCL appear to have altered thermoregulation as they have lower basal body temperature and are at increased risk of profound hypothermia during anesthesia [17]. In the present study, we sought to examine thermoregulation in an animal model of PPT1 deficiency, the *Ppt1*-knock out (*Ppt1*-KO) mouse, which has been shown to display several of the manifestations of INCL [18], [19]. In mice, brown adipose tissue has a pivotal role in thermogenesis and thermoregulation and unlike white adipose, is richly vascularized and has a high mitocondrial content. During cold exposure, there is upregulation of PGC-1α and uncoupling protein 1 (UCP1) in brown adipose tissue, which in turn generates heat, a process known as adaptive thermogenesis [20], [21], [22]. Given the previous reports of impaired adaptive energy metabolism and mitochondrial dysfunction in INCL and other neurodegenerative diseases [13], [14], we hypothesized that the *Ppt1*-KO mice would not upregulate PGC-1α and uncoupling protein 1 (UCP1) in brown adipose tissue and therefore would have impaired thermogenesis and thermoregulation.

**Figure 2:**
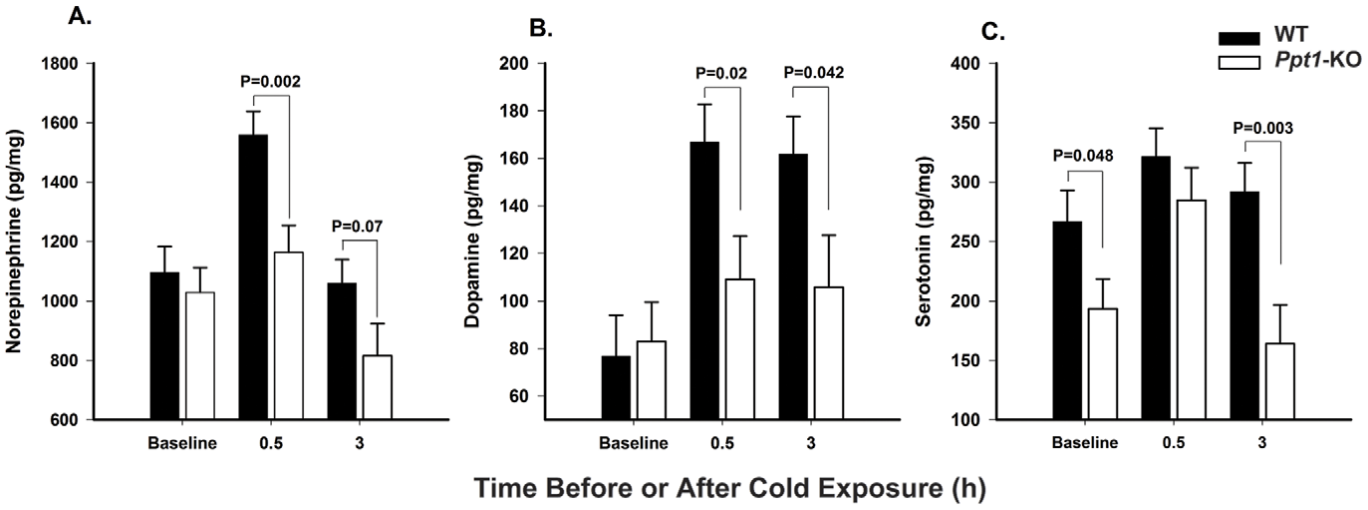
Neurotransmitter levels and β-adrenergic response to acute cold exposure in *Ppt1-*KO and wild-type mice. Bars represent mean (±SEM) brown adipose tissue levels of norepinephrine (A), dopamine (B), and serotonin (C) at baseline, after 0.5 h and 3 h of cold exposure in *Ppt1*-KO and wild-type control mice. Horizontal bars and respective p values show comparisons among groups indicated. Experiments were conducted in mice older than 20 weeks. Wild-type, N = 11, 13 and 7 for baseline, 0.5 h and 3 h cold challenge groups, respectively. *Ppt1*-KO, N = 13, 12 and 10 for baseline, 0.5 h and 3 h cold exposure groups, respectively.

## Materials and Methods

### Ethics Statement

The study protocol was approved by the Institutional Animal Care and Use Committees from the National Institutes of Health Clinical Center and the Children’s National Medical Center.

### Animals

We studied age-matched male and female B6.129-*Ppt^tm1Hof^* (*Ppt1*-KO) mice generated by gene targeting in embryonic stem cells [18] and backcrossed to C57BL/6J mice for 10 generations. As wild-type controls, we used C57BL/6J the background strain of *Ppt1*-KO mice. Animals were studied at young (8–9 weeks) and old (18–26 weeks) ages in order to obtain measurements during representative times of disease manifestations. In this model, at young age (8–9 weeks), *Ppt1*-KO mice show no overt neurologic deficits, none or very low level of neuronal autofluorescence and number of apoptotic bodies in brain [23]. In contrast, older (18–26 weeks) *Ppt1*-KO mice display neurologic deficits and widespread neurological pathology [19], as well as high levels of neuronal autofluorescence and apoptosis [23]. Animals had unrestricted access to food and water and were housed in 12-h light-dark cycles in a humidity controlled research animal facility with temperature set at 22.2°C.

**Figure 3:**
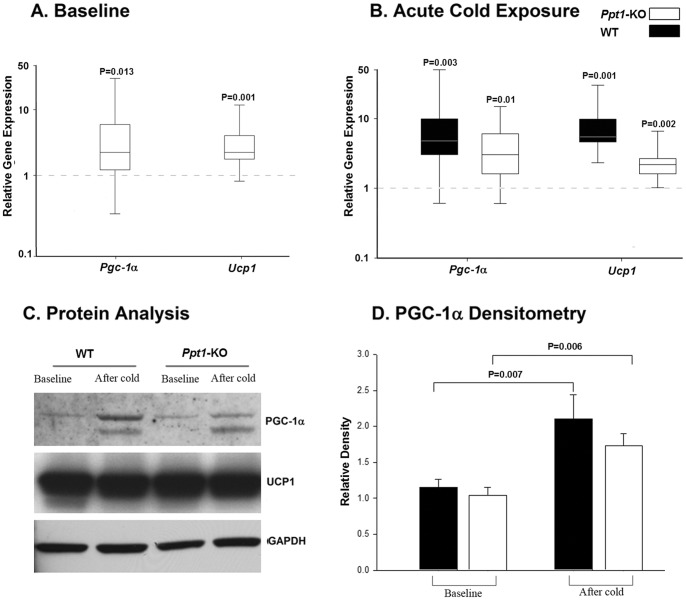
Cold-induced relative expression of *Pgc-1*α and *Ucp1* in brown adipose tissue *Ppt1-*KO and wild-type mice. A. Relative *Pgc1*α and *Ucp1* gene expressions at baseline comparing *Ppt1-*KO mice and wild-type (calibrator). The expression values for the wild-type controls were arbitrarily set to one for each gene examined and are indicated by the dashed horizontal line. B. Relative *Pgc1*α and *Ucp1* gene expressions in *Ppt1-*KO mice and wild-type controls after a six-hour cold (4°C) challenge compared with respective baseline expressions. The expression values for *Ppt1-*KO and wild-type mice at baseline were arbitrarily set to one for each gene examined and are indicated by the dashed horizontal line. Relative gene expressions were calculated using REST© version 2009 [25]. C. Representative western blots of brown adipose tissue extracts from mice at baseline conditions and after a 6-h cold (4°C) exposure, probed for PGC-1α, UCP1 and GAPDH. D. Relative density analysis of PGC-1α (+) bands, normalized to GAPDH levels, expressed in arbitrary units. In all panels, N = 4−6 for each group.

### Temperature Measurements and Thermoregulation Studies

#### Temperature measurements

Body temperature was measured serially using an electronic thermosensor weighing 120 mg (Bio Medic Data Systems, Seaford, Delaware) which was subcutaneously implanted caudad to the interscapular space at 4 weeks of age. Temperature measurements and cold and heat exposure experiments were conducted in the morning at the same time. Age-matched groups of wild-type and *Ppt1*-KO mice were studied simultaneously, except during the heat exposure experiments when groups of 5 animals were studied in consecutive days. During acute cold and heat exposures, mouse body temperature was measured every 30 min.

#### Acute cold and heat exposures

In order to evaluate the response to acute cold exposure, *Ppt1*-KO and wild-type animals were studied longitudinally both at young and older ages. Animals were individually housed in regular mouse cages and exposed to 4°C (in a cold room) for 6 hours (h) with unrestricted access to food and water. In order to evaluate the response to acute heat exposure, different cohorts of *Ppt1*-KO and wild-type animals were also studied longitudinally both at young and older ages. For those studies, animals were individually housed in regular mouse cages in an environmental chamber with temperature maintained at 38°C for 6 h with unrestricted access to food and water. As the heat environmental chamber could only house 5 cages at a time, wild-type and *Ppt1*-KO mice were studied during consecutive days. Technical difficulties prevented the recording of temperatures in the heat exposure experiments for young males. When these difficulties were corrected, animals were older and temperature measurements of young males undergoing heat challenge had not been collected.

**Figure 4:**
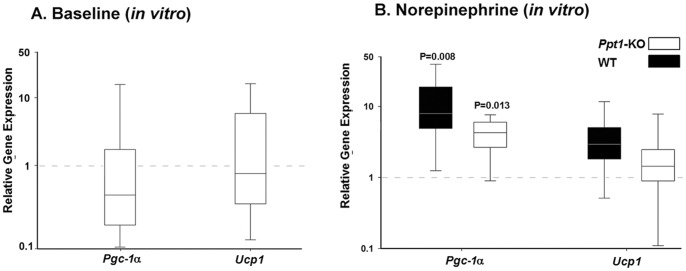
Brown adipose tissue response to norepinephrine in *Ppt1-*KO and wild-type mice. Brown adipose tissue explants from *Ppt1*-KO and wild-type animals were maintained in similar conditions for 3 days and subsequently exposed to 100 nM of norepinephrine for 3 hours. A. Baseline relative *Pgc1*α and *Ucp1* gene expressions in brown adipose tissue explants from *Ppt1-*KO mice and wild-type controls (calibrator). The expression values in brown adipose tissue of wild-type controls were arbitrarily set to one for each gene examined and are indicated by the dashed horizontal line. B. Relative *Pgc1*α and *Ucp1* gene expressions in *Ppt1-*KO mice and wild-type controls after a three-hour exposure to 100 nM of norepinephrine compared with respective baseline expressions. The expression values in brown adipose tissue of *Ppt1-*KO and wild-type mice before norepinephrine were arbitrarily set to one for each gene examined and are indicated by the dashed horizontal line. Relative gene expressions were calculated using REST© version 2009 [25]. In all panels, N = 5−6 for each group.

### In vitro Norepinephrine Stimulation of Brown Adipose Tissue

Brown adipose tissue from *Ppt1*-KO and WT mice (equal numbers of females and males in each group) was sliced at 50 µm, and placed on Millicell cell culture inserts (Millipore, Billerica, MA) in RPMI medium 1640, 10% FBS, and pen-strep (all from Life Technologies, Carlsbad, CA) in 24-well plates. Explants were incubated in media for 72 h and at that time, freshly prepared norepinephrine (100 nM, Sigma-Aldrich, St. Luis, MO) was added for 3 h. After a 3-h norepinephrine exposure, explants were frozen for RNA isolation and analyses.

**Figure 5:**
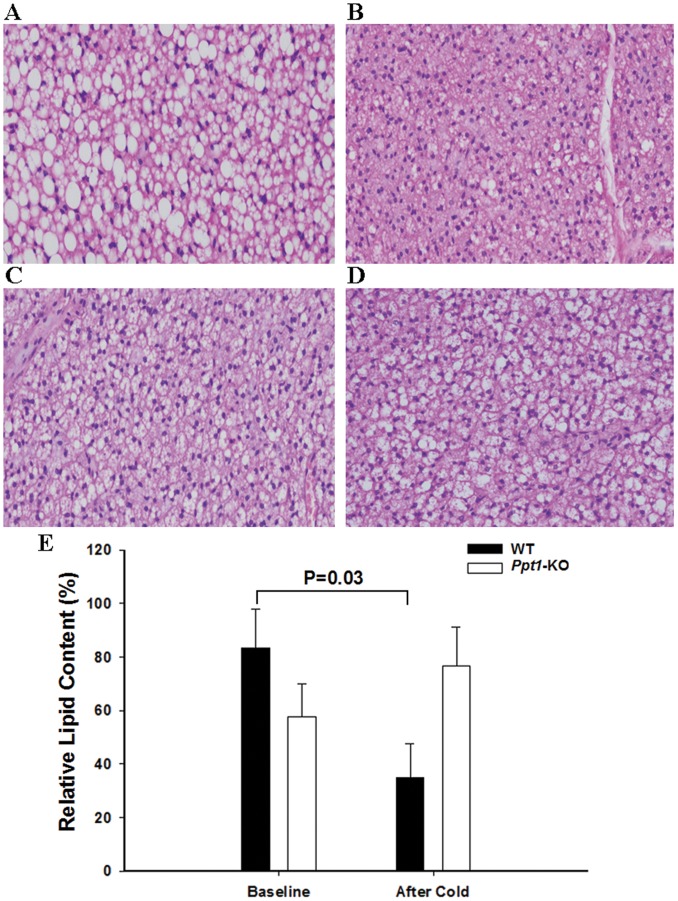
Fat content in brown adipose tissue during basal conditions and acute cold exposure. Representative 20× H&E staining of brown adipose tissue obtained before (A and C) and after (B and D) a 3-h acute cold exposure in *Ppt1-*KO (C and D) and wild-type (A and B) mice. E. Relative fat content in brown adipose tissue before and after a 3 h acute cold exposure in *Ppt1-*KO and wild-type mice. N = 3−5 in each group.

### Brown Adipose Tissue Neurotransmitter Levels

Two-hundred µl of 0.1 N perchloric acid were added to 30 mg of brown adipose tissue from *Ppt1*-KO and wild-type mice (equal numbers of females and males in each group). Each mixture was sonicated three times, 1 minute each with cooling on ice between the sonications. After vortexing, 100 µl of the samples were mixed with 50 µl of 10^−6^ M Me-5HT used as an internal standard. Samples were centrifuged at 14,000 rpm for 10 min (4°C), and 20 µl of supernatant were used for injection. Norepinephrine, dopamine, and serotonin concentrations were determined by using reverse-phase high-pressure liquid chromatography with electrochemical detection. The system consisted of an HPLC ESA 580 module, coupled to an ESA Coulochem II detector with an analytical electrochemical cell model 5014. Mobile Phase contained (per 1L) 1.8 g heptane sulfonic acid, 4.0 ml Phosphoric acid, 3.5 ml Triethylamine, 100 mg EDTA and 8.5% Acetonitrile. Column (Axxion, 5 micron, ODS C-18 4.6 mm i.d. X 25 cm) had a flow rate of 0.6 ml/min. The concentrations were calculated using a standard curve with high-purity norepinephrine, serotonin and dopamine standards.

### c-fos Expression

After the 6-h cold exposure, brains were dissected and fixed overnight in 4% paraformaldehyde in phosphate buffered saline (PBS). Tissue was cryoprotected by incubation in 30% sucrose in PBS with 0.1% NaN_3_ overnight. Brains were frozen in Tissue-Tek O.C.T compound (embedding medium for Optimal Cutting Temperature, Sakura Finetek USA Inc., Torrance, CA). Frozen coronal sections were cut at 5 µm thickness. Slides were air-dried at room temperature, rinsed three times in PBS with 0.01% Triton X-100 (PBS-T) and blocked with 2.5% normal goat serum. Slides were then incubated in PBS-T containing c-fos anti-serum (1∶20; Calbiochem, San Diego, CA) and 1% normal goat serum for 24 h at 4°C, rinsed three times in PBS-T, and incubated in fluorescent secondary antibody (FITC goat anti-rabbit IgG 1∶20, Invitrogen, Frederick, MD) for 1 h at room temperature. Slides were then cover-slipped using SlowFade Gold antifade reagent with DAPI (Invitrogen, Frederick, MD). The location of ventromedial hypothalamic nucleus was ascertained in DAPI-stained images at 10× magnification, using a mouse brain atlas as a guide [24]. The images were captured at the same exposure on a Zeiss Observer.Z1 fluorescent confocal microscope and analyzed using Photoshop 8.0. The rectangular region of interest of the same size (70 µm^2^) was used on each slide to count c-fos positive cells. For negative control we replaced the primary antibody with normal goat serum.

**Figure 6:**
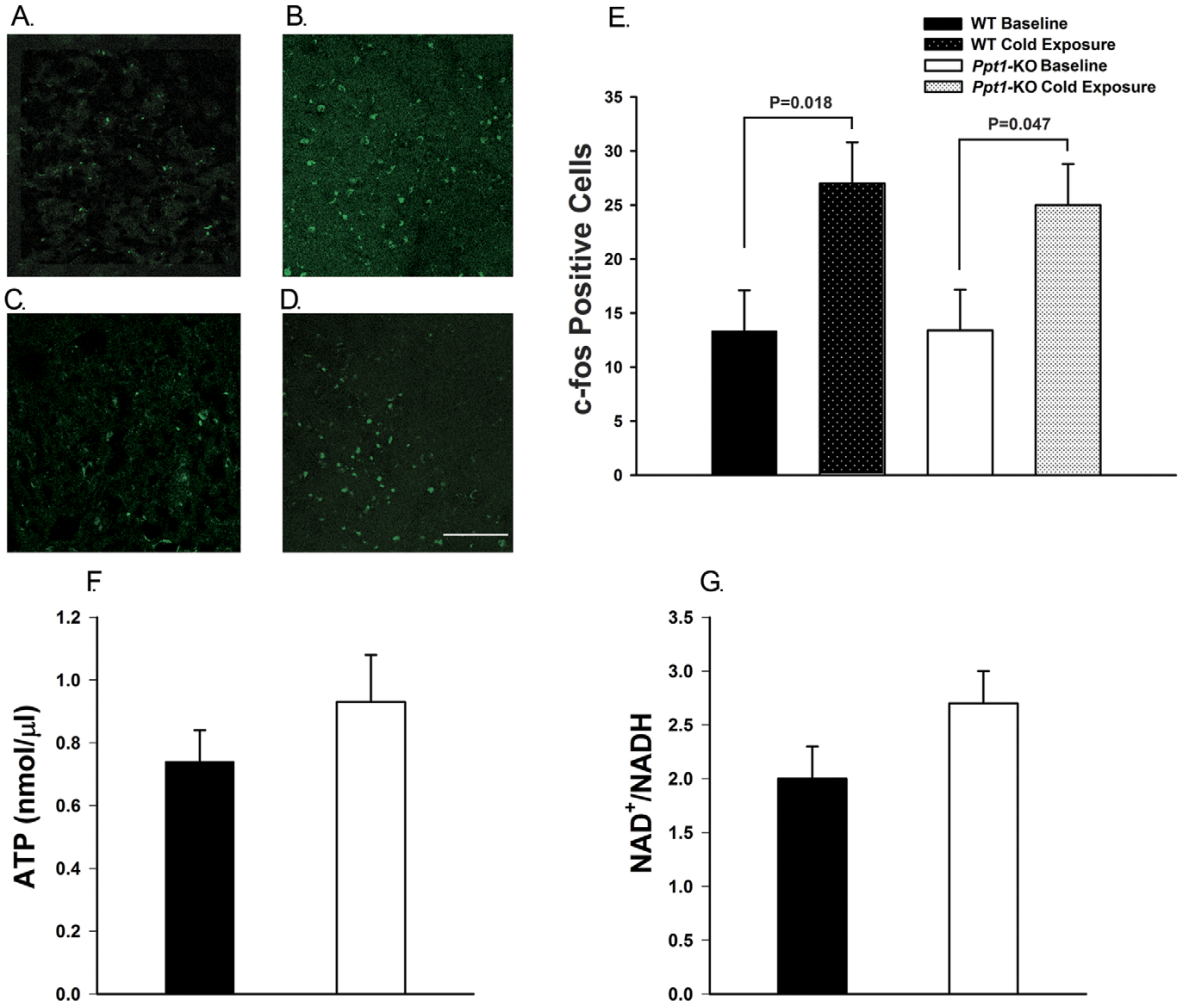
c-fos expression, ATP concentration and NAD^+^/NADH ratio in mouse brown adipose tissue. A–D. Representative images of brain c-fos immunostaining showing c-fos positive cells in ventromedial hypothalamic nucleus of wild-type (A and B) and *Ppt1*-KO (C and D) at baseline (A and C) and after cold exposure (B and D), N = 5−6 per group. E. Number of c-fos positive cells in ventromedial hypothalamic nucleus of *Ppt1*-KO and wild-type mice indicating that both wild-type and *Ppt1*-KO mice similarly upregulated *c-fos.* F. Baseline ATP concentration in brown adipose tissue of wild-type and *Ppt1*-KO mice are similar (N = 6 per group, p = NS). G. Baseline NAD^+^/NADH ratio in brown adipose tissue of wild-type and *Ppt1*-KO mice are similar (N = 6 per group, p = NS).

### NAD+/NADH and ATP Assays

Total NAD^+^ and NADH levels were determined using the NAD^+^/NADH Assay Kit (Abcam Inc., Cambridge, MA). Briefly, ∼20 mg of brown adipose tissue from 20-week-old animals were homogenized in 400 µl of extraction buffer and filtered using 10K cut-off filters. Half of the aliquot was heated at 60°C for 30 min to decompose NADP. Fifty µl of both native and heated samples were then added to a 96-well plate in duplicates and incubated in a cycling buffer for 5 min to convert NADP to NADPH. Color density after addition of 10 µl of the developer solution was read at 450 nm using an EnSpire 2300 (Perkin Elmer, Waltham, MD). NADP total (NADPt) and NADH levels were calculated based on a standard curve generated at the same time and the ratio was calculated using the formula (NADPt – NADPH)/NADPH.

Intracellular ATP was measured using a colorimetric/fluorometric assay kit (Abcam Inc., Cambridge, MA). Briefly, ∼ 20 mg of brown adipose tissue from 20-week-old animals were lysed in 200 µl of the ATP assay buffer and centrifuged at 15,000 g for 2 min. The supernatant was filtered through a 10K cut-off filter. Fifty µl of the resulting flow-through were added to a 96-well plate and followed by the addition of 50 µl of the Reaction Mix to each well. The absorbance was read at 550 nm using an EnSpire 2300 (Perkin Elmer, Waltham, MD). ATP content was calculated based on a standard curve generated at the same time.

### RNA Preparation and Real Time RT-PCR

RNA was extracted using the RNeasy mini kit (Qiagen, Valencia, CA) according to the manufacturer’s protocol. The quality of RNA preparation was assessed using a Bioanalyzer (Agilent, Santa Clara, CA). The concentration of RNA was determined using the NanoDrop (NanoDrop, Wilmington, DE).

Amplification reactions were performed in a mixture containing 10 µl of 2× TaqMan One Step PCR Master Mix, 0.5 µl of 40X Multiscribe and RNase Inhibitor Mix (all from Applied Biosystems, Foster City, CA) and 5 µl of target RNA (50 ng/µl). Each reaction contained either 1 µl of 20X Assays-on-Demand Gene Expression probe for murine *Pgc-1*α, *Ucp1*, β3 adrenergic receptor, actin or 0.6 µl of TaqMan GAPDH probe (all from Applied Biosystems). Total reaction mixture volume was 20 µl. No template controls were used for each probe to control for contamination. The PCR conditions included a cDNA synthesis step for 30 min at 48°C, followed by a polymerase activation step at 95°C for 10 min, and then by 40 cycles of 95°C for 15 s and 60°C for 60 s. The reactions were run on an ABI PRISM HT-7900 Sequence Detector (Applied Biosystems, Foster City, CA).

### Brown Adipose Tissue H&E Staining and Lipid Droplet Quantification

Brown adipose tissue was isolated and placed into 4% paraformaldehyde, 0.1 M sodium cacodylate buffer, 3% sucrose, pH 7.2. Tissue was paraffin-embedded, cut to 5 micron slices and mounted on glass slides. H&E staining was performed using standard methods by American Histo Labs Inc. (Gaithersburg, MD).

H&E slides were examined by one pathologist (MMQ) who was unaware of the mouse genotype as following: the entire surface area was evaluated for lipid content which was estimated as a percentage of clear areas relative to remaining areas of stained cellular components. The lipid content estimation for each sample was performed 5 different times over a 2 week period. Reproducibility of the lipid content measurements was confirmed over these 5 measurements.

### Statistical Methods

For physiologic experiments, we evaluated male and female groups separately. Given limitations on number of mice available, study groups included matching numbers of male and female mice and the data were pooled within groups for subsequent analyses.

For the physiologic experiments, analyses were done with SAS Version 9.1 software (SAS Institute, Inc, Cary, NC). For analyses comparing *Ppt1*-KO *vs*. wild-type mice, we used specialized methods for multivariate repeated measures ANOVA using PROC MIXED (for mixed models). For the basal temperature study the predictor variable of interest, genotype (*Ppt1*-KO *vs.* wild-type), gender, and age (young *vs.* old) were modeled as fixed effects; age was the repeated effect, modeled with compound symmetry covariance pattern, and mouse was a random effect (i.e., a “random intercept” model). For the acute cold and heat exposure studies, we used temperature changes from baseline as the outcome, and the predictor variables genotypes (*Ppt1*-KO *vs*. wild-type), gender, age (young *vs*. old), and time (over 6 h). For these studies temperature changes were repeated 12 times (every 30 minutes during 6 h) within the two ages. Here the covariance patterns were structured by two effects, by age and time. The first level structure across age was unstructured and the second-level structure across time was first-order autoregressive. Because of the small sample sizes, for all studies we used the Kenward and Roger method for computing the degrees of freedom for tests of fixed effects, and all models included interaction terms. The value of the estimator (“least square means” in SAS terminology) and associated standard error of the estimator and P values are reported.

Group-wise comparisons of gene expression ratio measured by RT-PCR were calculated after normalization to endogenous GAPDH and were expressed relative to respective controls (normalized to 1) using the comparative Ct method and the relative expression software tool REST© version 2009 [25]. The comparisons of concentrations of norepinephrine, serotonin and dopamine were performed using two way analysis of variance (2-way ANOVA) and pair-wise multiple comparisons using Holm-Sidak method (SigmaPlot 11.0). Two-sided p<0.05 was considered to be statistically significant.

## Results

As previously described, by the age of 20 weeks [18], all *Ppt1*-KO animals had developed abnormal gait, rough and ungroomed appearance, myoclonic jerks, and skin lesions.

### PPT1 Deficiency is Associated with Decreases in Basal Temperature

Overall, *Ppt1*-KO mice had significantly lower basal temperatures compared to wild-type animals (p = 0.006) in a manner that varied with age and sex (Figure 1A). At the younger age (8–9 weeks), females (p = 0.4) and males (p = 0.9) *Ppt1*-KO compared with wild-type mice had similar basal temperatures. In contrast, at 18–26 weeks, both female (p = 0.003) and male (p = 0.007) *Ppt1*-KO animals had lower basal temperatures compared to female and male wild-type of a similar age. In addition, with aging (from 8–9 to 18–26 weeks) male and female *Ppt1*-KO mice combined developed significant (p = 0.009) decreases in basal temperature (Figure 1A). Conversely, in wild-type mice there were no significant changes in basal temperature comparing male and female animals or age (8–9 to 18–26 weeks) groups (both sex and age effects, p>0.22).

### PPT1 Deficiency is Associated with Altered Response to Acute Cold Exposure

When acutely exposed to cold temperatures, *Ppt1*-KO and wild-type mice displayed similar behavior and intermittently shivered. Overall, with a 6-h cold exposure both *Ppt1*-KO and wild-type animals had significant decreases in body temperature (Figure 1B, both p<0.0001) that varied by genotype, age, and sex. With acute cold exposure, at the young age (8–9 weeks), both females (p = 0.64) and males (p = 0.93) *Ppt1*-KO and wild-type mice had similar significant mild decreases (all p<0.0001) from baseline body temperatures (Figure 1B). Interestingly, with acute cold exposure at the older age (18–26 weeks) both female (p = 0.003) and male (p = 0.03) *Ppt1*-KO mice had greater decreases from baseline body temperatures compared to female and male wild-type mice respectively (Figure 1B). In addition, over a 6-h exposure to cold temperatures at the older age, females compared to males both in *Ppt1*-KO and wild-type mice had greater decreases from baseline body temperature (both p<0.0001 female *vs.* male *Ppt1*-KO and wild-type mice, Figure 1B).

In addition, with acute cold exposure *Ppt1*-KO and wild-type female mice at the older age (18–26 weeks) compared to when they were younger (8–9 weeks) had greater decreases from baseline body temperature (p<0.0001 for *Ppt1*-KO and p = 0.003 for wild-type animals). In contrast, *Ppt1*-KO and wild-type males had lesser decreases in body temperature when exposed to cold at older age compared to when they were younger (p = 0.0007 for *Ppt1*-KO and p<0.0001 for wild-type animals).

### PPT1 Deficiency is Associated with Altered Response to Acute Heat Exposure

During acute heat exposure, *Ppt1*-KO and wild-type mice showed similar behavior patterns and decreased physical activity. Overall, with an acute 6-h heat exposure, *Ppt1*-KO and wild-type animals had significant increases in body temperature from baseline (p = 0.004) that varied by age and sex (Figure 1C). At the older age, combining male and female, *Ppt1*-KO mice had greater increases in body temperature compared to wild-type animals (p = 0.048). When females were compared at older and younger ages, for both genotypes the increase in temperature was greater at the older age (*Ppt1*-KO P = 0.02, wild-type p = 0.004, Figure 1C). With heat exposure at younger (8–9 weeks) and older (18–26 weeks) ages, *Ppt1*-KO and wild-type female mice had similar (both ages p>0.2) significant increases in body temperature compared to baseline (all p<0.0001, Figure 1C).

### PPT1 Deficiency is Associated with Alteration in Neurotransmitter Levels and β-adrenergic Response to Acute Cold Exposure

In order to examine the sympathetic nervous system and serotonergic neurons activation in brown adipose tissue, we measured tissue levels of norepinephrine, dopamine, and serotonin before and after (0.5 and 3 h) cold exposure in *Ppt1*-KO and wild-type mice. At baseline, *Ppt1*-KO mice had similar (p = NS) norepinephrine and dopamine and significantly (p = 0.013) lower serotonin levels in brown adipose tissue compared with wild-type controls (Figure 2). Over a 3-h cold exposure *Ppt1*-KO mice had significantly different changes in norepinephrine (p = 0.002), dopamine (p = 0.016), and serotonin (p<0.001) levels in brown adipose tissue compared to wild-type controls. Specifically, compared with baseline, wild-type animals had significant increases in brown adipose tissue norepinephrine levels by 0.5 h (p<0.001) which then significantly decreased (p<0.001) and returned to baseline levels by 3 h. In contrast, compared with baseline, *Ppt1*-KO had non-significant changes by 0.5 h (p = 0.28) and then significant decreases (p = 0.049) in norepinephrine brown adipose tissue levels by 3-h of cold exposure (Figure 2A). With regards to dopamine, compared with baseline, wild-type animals had significant increases at 0.5 h (p<0.001) and 3 h (p = 0.001) whereas, *Ppt1*-KO had no significant changes in brown adipose tissue dopamine levels with acute cold exposure (Figure 2B). Lastly, wild-type animals had no significant changes in brown adipose tissue serotonin levels, whereas *Ppt1*-KO, compared with baseline, had significant increases by 0.5 h (p = 0.034) and then decreases by 3 h (p = 0.019) in serotonin brown adipose tissue levels during acute cold exposure (Figure 2C).

We then examined mRNA levels of β3 adrenergic receptor in brown adipose tissue, the receptor involved in brown adipose tissue thermogenesis. We found that both at baseline and after acute cold exposure *Ppt1*-KO and wild-type animals had similar β3 adrenergic receptor mRNA levels in brown adipose tissue (p = NS, data not shown).

### Brown Adipose Tissue Thermogenesis in the *Ppt1*-KO Mouse

We then examined the expression of *Pgc-1α* and *Ucp1* in brown adipose tissue during baseline conditions and after a 6-h acute cold exposure in 20-week-old *Ppt1*-KO and wild-type mice (Figure 3). At baseline (18 to 26 weeks) *Ppt1*-KO mice had significantly higher mRNA levels of *Pgc-1α* (p = 0.013) and *Ucp1* (p = 0.001) in brown adipose tissue compared with wild-type animals (Figure 3A). It is noteworthy that at 20 weeks, *Ppt1*-KO mice had lower body temperature compared with wild-type animals (Figure 1A). Surprisingly, after a 6-h acute cold exposure in 20-week-old animals, both wild-type and *Ppt1*-KO mice had comparable significant upregulation of both *Pgc-1α* and *Ucp1* compared with respective baseline (all p≤0.01, Figure 3B). Western blot analysis (Figures 3C and 3D) showed that at baseline, *Ppt1*-KO and wild-type mice had similar levels of PGC-1α and UCP1 in brown adipose tissue and after acute cold exposure, both *Ppt1*-KO (p = 0.006) and wild-type (p = 0.007) mice had significant increases in PGC-1α protein levels compared to respective baseline levels (Figure 3D).

We also examined the *in vitro* response of *Ppt*1-KO and wild-type brown adipose tissue explants exposed to the same temperature and to a 3-h incubation with 100 nM of norepinephrine (Figure 4A and 4B respectively). After 3-days in culture, brown adipose tissue explants from *Ppt1*-KO mice had similar mRNA levels of *Pgc-1α* and *Ucp1* (p = NS). After a 3-h incubation with 100 nM of norepinephrine, brown adipose tissue from wild-type and *Ppt1*-KO mice had significant upregulation of *Pgc-1α* compared to respective baseline expression (p = 0.008 for wild-type and p = 0.013 for *Ppt1*-KO mice, Figure 4B). Compared with baseline expressions, 3-h incubation with norepinephrine yielded no significant changes in *Ucp1* mRNA levels compared to baseline in *Ppt1*-KO and wild-type mice.

### PPT1 Deficiency is Associated with Alterations in Brown Adipose Tissue Morphology

In *Ppt1*-KO animals, during basal conditions, brown fat appeared to be more oxyphilic and had less vacuolization compared to wild-type animals. Three hours of acute cold exposure yielded morphologic changes in brown adipose tissue which were significantly different comparing *Ppt1*-KO and wild-type mice. Specifically, after a 3-h cold exposure, brown fat in wild-type animals changed morphology in that there were significant decreases in overall degree of vacuolization (lipid droplets) and increases in oxyphilic morphology. In contrast, in *Ppt1*-KO mice there were no significant changes in brown fat tissue morphology (Figure 5). We then quantified the overall lipid droplets content in brown adipose tissue before and after cold exposure. After a 3-h cold exposure, there were significant changes in lipid droplet content that varied according to genotype (p = 0.032). Specifically, after cold exposure, wild-type animals had significant decreases (p = 0.03) whereas *Ppt1*-KO had no significant changes in lipid droplet content in brown adipose tissue compared with baseline measurements.

### c-fos Expression in Ventromedial Hypothalamic Nucleus of *Ppt1*-KO Mice

In order to examine neuronal activation after acute cold exposure, we measured changes in c-fos expression in the ventromedial hypothalamic nucleus in *Ppt1*-KO and wild-type animals. The number of c-fos positive neurons noted at baseline was similar comparing wild-type and *Ppt1*-KO animals (p = NS, Figure 6A and 6C). After cold exposure, both wild-type (Figure 6B) and *Ppt1*-KO (Figure 6D) animals significantly upregulated c-fos expression (p = 0.018 and p = 0.047 for wild-type and *Ppt1*-KO respectively, Figure 6E).

### ATP Levels and NAD+/NADH Ratios in Brown Adipose Tissue of *Ppt1*-KO and Wild-type Mice

In order to evaluate mitochondrial function, we measured ATP levels and NAD+/NADH ratios during basal conditions in 5-month-old animals. *Ppt1*-KO and wild-type mice had similar levels of ATP (Figure 6F) and NAD+/NADH ratios (Figure 6G) in brown adipose tissue at baseline (p = NS, Figure 6).

## Discussion

In the present investigation we evaluated thermoregulation in the *Ppt1*-KO mouse, a model that recapitulates neurodegenerative and pathological features of human INCL. We found that these animals have thermoregulation defects akin to what has been previously reported in children with INCL [17]. Specifically, with aging, *Ppt1*-KO mice drop basal body temperature and during acute cold exposure, these mice develop hypothermia compared with wild-type controls. During acute cold stress, *Ppt1*-KO mice show activation of hypothalamic neurons as shown by increases in c-fos. However, despite evidence of neuronal activation in hypothalamus during acute cold exposure, brown adipose tissue levels of norepinephrine and dopamine in *Ppt1*-KO are lower than those in wild-type animals. Moreover, *Ppt1*-KO mice, unlike wild-type animals, show no decreases in brown adipose tissue lipid droplets, suggesting a defect in lipid handling during acute cold exposure stress. Further, contrary to our hypothesis, during cold exposure *Ppt1*-KO mice appropriately upregulate mRNA and protein levels of *Pgc-1α* and *Ucp1* in brown adipose tissue. Taken together, these findings suggest that *Ppt1*-KO mice have impaired thermoregulation and altered lipid handling in brown adipose tissue that likely results from decreased tissue levels of neurotransmitters.

After we determined alterations in the thermoregulation phenotype in *Ppt1*-KO mice, we hypothesized that brown adipose tissue thermogenesis would be impaired because of alterations in cellular metabolism and impairment in PGC-1α regulation and mitochondria dysfunction known to occur in INCL. This hypothesis would be in keeping with our previously reported findings that in fibroblasts from INCL patients and in brain from *Ppt1*-KO mice, levels of NAD^+^/NADH ratio and PGC-1α are down regulated [13]. Here we show that with cold exposure, *Ppt1*-KO mice appropriately upregulated brown adipose tissue gene expression of *Pgc-1*α as well as *Ucp*1. While we saw an increase in PGC-1α protein levels, the increases in *Ucp1* mRNA levels were not accompanied by corresponding increases in protein levels [26]. This finding is expected and given that UCP1 has a 5-day half-life, one would only see corresponding changes in protein levels after approximately 10 days [26]. In addition, these *in vivo* findings during acute cold exposure were also observed *in vitro* when brown adipocytes from *Ppt1*-KO mice appropriately upregulated *Pgc-1α* in response to norepinephrine. Further in brown adipose tissue, NAD^+^/NADH ratios and ATP levels were similar in wild-type and *Ppt1*-KO mice suggesting intact mitochondrial function. Therefore, our *in vivo* and *in vitro* findings disprove our hypothesis. Thus, taken together, our present and previous findings [13] suggest that the expression of PGC-1α in *Ppt1*-KO mice varies according to the tissue examined and to the type of metabolic demand to which animals are exposed.

In mammals, once cold temperatures are sensed, hypothalamic neurons are activated and sympathetic tone increases [27], [28], [29], [30]. In brown adipose tissue, sympathetic innervation has been amply demonstrated and its activation has a pivotal role in thermogenesis during cold exposure [31]. Here we show that both wild type and *Ppt1*-KO mice similarly upregulated c-fos in hypothalamus suggesting that mice of both genotypes similarly sensed low temperatures and activated hypothalamic neurons. This finding is in concert with reports that the hypothalamus is one of the least affected areas in *Ppt1*-KO mouse brains [19]. However, it is surprising that downstream from hypothalamic activation, *Ppt1*-KO mice had lower levels of norepinephrine and dopamine in brown adipose tissue after acute cold exposure, suggesting that an abnormality in the autonomic effectors could contribute to hypothermia in *Ppt1*-KO mice. Interestingly, while *Ppt1*-KO mice do not show increases in norepinephrine and dopamine levels in brown adipose tissue, they show transient increases in serotonin tissue levels after 0.5 h of cold exposure; a change that was not observed in wild-type animals. Researchers have also shown that during acute cold exposure, serotonergic neurons have a role in thermogenesis possibly by increasing sympathetic nerve activity in brown adipose tissue [32], [33]. Taken together, our findings suggest that PPT1 deficiency might be associated with perturbations in serotonergic neurons and sympathetic nervous system functions which in turn might impair temperature regulations during cold exposure.

During acute cold exposure, activation of the sympathetic nervous system, release of norepinephrine, and activation of β3 adrenergic receptors [21], [22], [34] triggers the lysis of lipid droplets in brown adipose tissue and release of free fatty acids. In turn, some of those fatty acids activate UCP1 and others are used as substrate for thermogenesis activity [21], [22], [34], [35], [36]. Researchers have shown that cold exposure in rodents is associated with morphologic changes that correspond to lipid droplets lipolysis in brown adipose tissue [37], [38], [39]. Here we found that after 3-h cold exposures, Ppt1-KO mice, unlike wild-type animals, do not display such morphologic changes, which might suggest that lipolysis of lipid droplets in *Ppt1*-KO mice was impaired. Therefore, these findings are suggestive of a model whereby during acute cold exposure, *Ppt1*-KO mice increase thermogenesis capacity by upregulating *Ucp1*, but not necessarily thermogenesis activity as lipolysis is diminished and substrate for thermogenesis is incompletely available. As a result, *Ppt1*-KO mice are unable to maintain temperature during cold stress.

Researchers have shown that PPT1 deficiency is associated with altered energy balance and lipid metabolism [13], [40], [41]. *Ppt1*-KO mice have decreased adiposity despite having increased food intake and reduced metabolic rate compared with control animals [41]. In addition, others have also shown that *Ppt1*-KO mice have disturbances in cholesterol metabolism and calcium homeostasis before synaptic dysfunction ensues [40]. Those studies were conducted during baseline conditions. Here we show that during acute cold exposure, a known stressful condition that is associated with increased metabolic demands, *Ppt1*-KO mice are unable to meet these metabolic demands as suggested by absence of lipolysis in brown adipose tissue and inability to maintain body temperature during cold exposure. Therefore our findings add to the existing body of literature suggesting that PPT1 deficiency is associated with altered energy balance which not only could contribute to neurodegeneration but also to impairment in thermoregulation.

We must consider some limitations of our study. We cannot rule out the possibility that *Ppt1*-KO mice were unable to regulate temperature because of impaired shivering thermogenesis. While we did not quantitate shivering thermogenesis, we know that during cold exposure, these animals qualitatively shiver as wild-type animals do. Another limitation is that our cold exposure only lasted 6 hours and some might argue that this is not enough time to appropriately evaluate non-shivering thermogenesis [21]. Therefore we cannot comment on the outcome of prolonged cold exposure. However, given our findings after a 3- and 6-h cold exposure, we hypothesize that a longer cold challenge would further decrease body temperature given the known metabolic alteration in *Ppt1*-KO mice. The findings that *Ppt1*-KO mice, unlike wild-type animals, do not display the morphologic changes suggestive of lipolysis with cold exposure [37], [38], [39] should be interpreted with caution as the use of histologic sections to quantitate lipid content in brown adipose tissue carries its limitations. However, despite these limitations, our findings of altered response of brown adipose tissue to cold stress in *Ppt1*-KO mice suggest that PPT1 might be important for the response to cold stress and brown fat metabolism and thermogenesis.

In summary, we show that PPT1 deficiency is associated with impairments in thermoregulation and neurotransmitter release and lipolysis in brown adipose tissue. While one must be circumspect about extrapolating findings in rodents to humans, it is noteworthy that we have previously shown that patients with INCL are at increased risk for profound hypothermia during anesthesia. Therefore, extrapolating the findings of this investigation clinically, temperature regulation is likely to be a problem in patients with INCL and as such, special care has to be taken to prevent hypothermia in these patients. Further, our findings raise hypotheses that warrant further testing to improve our understanding of the potential role of lipid handling impairment in the setting of PPT1 deficiency and in the pathobiology of INCL.

## References

[1] Haltia M (2006) The neuronal ceroid-lipofuscinoses: from past to present. Biochim Biophys Acta 1762: 850–856. 16908122 10.1016/j.bbadis.2006.06.010

[2] Santavuori P, Lauronen L, Kirveskari E, Aberg L, Sainio K, et al (2000) Neuronal ceroid lipofuscinoses in childhood. Neurol Sci 21: S35–41. 11073226 10.1007/s100720070038

[3] Santavuori P, Lauronen L, Kirveskari K, Aberg L, Sainio K (2000) Neuronal ceroid lipofuscinoses in childhood. Suppl Clin Neurophysiol 53: 443–451. 12741032 10.1016/s1567-424x(09)70193-x

[4] Goebel HH, Wisniewski KE (2004) Current state of clinical and morphological features in human NCL. Brain Pathol 14: 61–69. 14997938 10.1111/j.1750-3639.2004.tb00499.x PMC8095868

[5] Vesa J, Hellsten E, Verkruyse LA, Camp LA, Rapola J, et al (1995) Mutations in the palmitoyl protein thioesterase gene causing infantile neuronal ceroid lipofuscinosis. Nature 376: 584–587. 7637805 10.1038/376584a0

[6] Camp LA, Verkruyse LA, Afendis SJ, Slaughter CA, Hofmann SL (1994) Molecular cloning and expression of palmitoyl-protein thioesterase. J Biol Chem 269: 23212–23219. 7916016

[7] Hofmann SL, Peltonen L (2001) The neuronal ceroid lipofuscinosis. In: Scriver CR, Sly WS, Childs B, editors. The Metabolic & Molecular Bases of Inherited Disease. New York: McGraw-Hill. 3877–3894.

[8] Kim SJ, Zhang Z, Lee YC, Mukherjee AB (2006) Palmitoyl-protein thioesterase-1 deficiency leads to the activation of caspase-9 and contributes to rapid neurodegeneration in INCL. Hum Mol Genet 15: 1580–1586. 16571600 10.1093/hmg/ddl078

[9] Kim SJ, Zhang Z, Hitomi E, Lee YC, Mukherjee AB (2006) Endoplasmic reticulum stress-induced caspase-4 activation mediates apoptosis and neurodegeneration in INCL. Hum Mol Genet 15: 1826–1834. 16644870 10.1093/hmg/ddl105

[10] Wei H, Kim SJ, Zhang Z, Tsai PC, Wisniewski KE, et al (2008) ER and oxidative stresses are common mediators of apoptosis in both neurodegenerative and non-neurodegenerative lysosomal storage disorders and are alleviated by chemical chaperones. Hum Mol Genet 17: 469–477. 17989065 10.1093/hmg/ddm324

[11] Zhang Z, Lee YC, Kim SJ, Choi MS, Tsai PC, et al (2007) Production of lysophosphatidylcholine by cPLA2 in the brain of mice lacking PPT1 is a signal for phagocyte infiltration. Hum Mol Genet 16: 837–847. 17341491 10.1093/hmg/ddm029

[12] Kim SJ, Zhang Z, Sarkar C, Tsai PC, Lee YC, et al (2008) Palmitoyl protein thioesterase-1 deficiency impairs synaptic vesicle recycling at nerve terminals, contributing to neuropathology in humans and mice. J Clin Invest 118: 3075–3086. 18704195 10.1172/JCI33482 PMC2515381

[13] Wei H, Zhang Z, Saha A, Peng S, Chandra G, et al (2011) Disruption of adaptive energy metabolism and elevated ribosomal p-S6K1 levels contribute to INCL pathogenesis: partial rescue by resveratrol. Hum Mol Genet 20: 1111–1121. 21224254 10.1093/hmg/ddq555 PMC3043662

[14] Weydt P, Pineda VV, Torrence AE, Libby RT, Satterfield TF, et al (2006) Thermoregulatory and metabolic defects in Huntington’s disease transgenic mice implicate PGC-1alpha in Huntington’s disease neurodegeneration. Cell Metab 4: 349–362. 17055784 10.1016/j.cmet.2006.10.004

[15] Geisler S, Holmstrom KM, Skujat D, Fiesel FC, Rothfuss OC, et al (2010) PINK1/Parkin-mediated mitophagy is dependent on VDAC1 and p62/SQSTM1. Nat Cell Biol 12: 119–131. 20098416 10.1038/ncb2012

[16] Querfurth HW, LaFerla FM (2010) Alzheimer’s disease. N Engl J Med 362: 329–344. 20107219 10.1056/NEJMra0909142

[17] Miao N, Levin SW, Baker EH, Caruso RC, Zhang Z, et al (2009) Children with infantile neuronal ceroid lipofuscinosis have an increased risk of hypothermia and bradycardia during anesthesia. Anesth Analg 109: 372–378. 19608805 10.1213/ane.0b013e3181aa6e95 PMC2743022

[18] Gupta P, Soyombo AA, Atashband A, Wisniewski KE, Shelton JM, et al (2001) Disruption of PPT1 or PPT2 causes neuronal ceroid lipofuscinosis in knockout mice. Proc Natl Acad Sci U S A 98: 13566–13571. 11717424 10.1073/pnas.251485198 PMC61081

[19] Bible E, Gupta P, Hofmann SL, Cooper JD (2004) Regional and cellular neuropathology in the palmitoyl protein thioesterase-1 null mutant mouse model of infantile neuronal ceroid lipofuscinosis. Neurobiol Dis 16: 346–359. 15193291 10.1016/j.nbd.2004.02.010

[20] Liang H, Ward WF (2006) PGC-1alpha: a key regulator of energy metabolism. Adv Physiol Educ 30: 145–151. 17108241 10.1152/advan.00052.2006

[21] Cannon B, Nedergaard J (2011) Nonshivering thermogenesis and its adequate measurement in metabolic studies. J Exp Biol 214: 242–253. 21177944 10.1242/jeb.050989

[22] Cannon B, Nedergaard J (2004) Brown adipose tissue: function and physiological significance. Physiol Rev 84: 277–359. 14715917 10.1152/physrev.00015.2003

[23] Zhang Z, Lee YC, Kim SJ, Choi MS, Tsai PC, et al (2006) Palmitoyl-protein thioesterase-1 deficiency mediates the activation of the unfolded protein response and neuronal apoptosis in INCL. Hum Mol Genet 15: 337–346. 16368712 10.1093/hmg/ddi451

[24] Franklin KBJ, Paxinos G (2008) The mouse brain in stereotaxic coordinates. New York: Elsevier Inc. 360 p.

[25] Pfaffl MW, Horgan GW, Dempfle L (2002) Relative expression software tool (REST) for group-wise comparison and statistical analysis of relative expression results in real-time PCR. Nucleic Acids Res 30: e36. 11972351 10.1093/nar/30.9.e36 PMC113859

[26] Nedergaard J, Golozoubova V, Matthias A, Asadi A, Jacobsson A, et al (2001) UCP1: the only protein able to mediate adaptive non-shivering thermogenesis and metabolic inefficiency. Biochim Biophys Acta 1504: 82–106. 11239487 10.1016/s0005-2728(00)00247-4

[27] Morrison SF, Nakamura K, Madden CJ (2008) Central control of thermogenesis in mammals. Exp Physiol 93: 773–797. 18469069 10.1113/expphysiol.2007.041848 PMC2496891

[28] Nakamura K, Morrison SF (2007) Central efferent pathways mediating skin cooling-evoked sympathetic thermogenesis in brown adipose tissue. Am J Physiol Regul Integr Comp Physiol 292: R127–136. 16931649 10.1152/ajpregu.00427.2006 PMC2441894

[29] Nakamura K, Morrison SF (2008) A thermosensory pathway that controls body temperature. Nat Neurosci 11: 62–71. 18084288 10.1038/nn2027 PMC2423341

[30] Nakamura K, Morrison SF (2008) Preoptic mechanism for cold-defensive responses to skin cooling. J Physiol 586: 2611–2620. 18388139 10.1113/jphysiol.2008.152686 PMC2464333

[31] Bartness TJ, Vaughan CH, Song CK (2010) Sympathetic and sensory innervation of brown adipose tissue. Int J Obes (Lond) 34 Suppl 1S36–42. 20935665 10.1038/ijo.2010.182 PMC3999344

[32] Madden CJ, Morrison SF (2010) Endogenous activation of spinal 5-hydroxytryptamine (5-HT) receptors contributes to the thermoregulatory activation of brown adipose tissue. Am J Physiol Regul Integr Comp Physiol 298: R776–783. 20071609 10.1152/ajpregu.00614.2009 PMC2838665

[33] Cummings KJ, Hewitt JC, Li A, Daubenspeck JA, Nattie EE (2011) Postnatal loss of brainstem serotonin neurones compromises the ability of neonatal rats to survive episodic severe hypoxia. J Physiol 589: 5247–5256. 21911619 10.1113/jphysiol.2011.214445 PMC3225677

[34] Matthias A, Ohlson KB, Fredriksson JM, Jacobsson A, Nedergaard J, et al (2000) Thermogenic responses in brown fat cells are fully UCP1-dependent. UCP2 or UCP3 do not substitute for UCP1 in adrenergically or fatty scid-induced thermogenesis. J Biol Chem 275: 25073–25081. 10825155 10.1074/jbc.M000547200

[35] Shabalina IG, Jacobsson A, Cannon B, Nedergaard J (2004) Native UCP1 displays simple competitive kinetics between the regulators purine nucleotides and fatty acids. J Biol Chem 279: 38236–38248. 15208325 10.1074/jbc.M402375200

[36] Nedergaard J, Bengtsson T, Cannon B (2011) New powers of brown fat: fighting the metabolic syndrome. Cell Metab 13: 238–240. 21356513 10.1016/j.cmet.2011.02.009

[37] Thomsom JF, Habeck DA, Nance SL, Beetham KL (1969) Ultrastructural and biochemical changes in brown fat in cold-exposed rats. J Cell Biol 41: 312–334. 4304742 10.1083/jcb.41.1.312 PMC2107720

[38] Carter EA, Bonab AA, Hamrahi V, Pitman J, Winter D, et al (2011) Effects of burn injury, cold stress and cutaneous wound injury on the morphology and energy metabolism of murine brown adipose tissue (BAT) in vivo. Life Sci 89: 78–85. 21565200 10.1016/j.lfs.2011.04.014 PMC4081480

[39] Bartelt A, Bruns OT, Reimer R, Hohenberg H, Ittrich H, et al (2011) Brown adipose tissue activity controls triglyceride clearance. Nat Med 17: 200–205. 21258337 10.1038/nm.2297

[40] Ahtiainen L, Kolikova J, Mutka AL, Luiro K, Gentile M, et al (2007) Palmitoyl protein thioesterase 1 (Ppt1)-deficient mouse neurons show alterations in cholesterol metabolism and calcium homeostasis prior to synaptic dysfunction. Neurobiol Dis 28: 52–64. 17656100 10.1016/j.nbd.2007.06.012

[41] Woloszynek JC, Coleman T, Semenkovich CF, Sands MS (2007) Lysosomal dysfunction results in altered energy balance. J Biol Chem 282: 35765–35771. 17911106 10.1074/jbc.M705124200

